# Sex and inflammation in respiratory diseases: a clinical viewpoint

**DOI:** 10.1186/2042-6410-4-16

**Published:** 2013-09-01

**Authors:** Georges J Casimir, Nicolas Lefèvre, Francis Corazza, Jean Duchateau

**Affiliations:** 1 Department of Pulmonology, Allergology and Cystic Fibrosis, Hôpital Universitaire des Enfants Reine Fabiola, Avenue JJ. Crocq 15, B-1020, Brussels, Belgium; 2 Laboratory of Immunology, Hôpital Universitaire Brugmann, Place Arthur Van Gehuchten, 4, B-1020, Brussels, Belgium; 3 Laboratory of Pediatrics, Université Libre de Bruxelles (ULB), Place Arthur Van Gehuchten 4, B-1020, Brussels, Belgium

**Keywords:** Sex, Respiratory inflammation, X chromosome, Hormones, Cytokines

## Abstract

This review discusses sex differences in the prognosis of acute or chronic inflammatory diseases. The consequences of severe inflammation vary in relation to sex, depending on illness duration. In the majority of acute diseases, males present higher mortality rates, whereas continuous chronic inflammation associated with tissue damage is more deleterious in females. The recruitment of cells, along with its clinical expression, is more significant in females, as reflected by higher inflammatory markers. Given that estrogens or androgens are known to modulate inflammation, their different levels in males and females cannot account for the sexual dimorphism observed in humans and animals from birth to death with regard to inflammation. Numerous studies evaluated receptors, cytokine production, and clinical outcomes in both animals and humans, revealing that estrogens clearly modulate the immune response, but the results are contradictory and difficult to link to hormone concentrations. Even in prepubescent children, the presentation of acute pneumonia or chronic diseases mimics the adult pattern. Several genes located on the X chromosome have been shown to encode molecules involved in inflammation. Moreover, 10% to 15% of the genes from silenced X chromosome may escape inhibition. Females are also a mosaic of cells with genes from either paternal or maternal X chromosome. Therefore, polymorphism of X-linked genes would result in the presence of two cell populations with distinct regulatory arsenals, providing females with greater diversity to fight against infectious challenges, in comparison with the uniform cell populations in hemizygous males. The similarities observed between males and Turner syndrome patients using an endotoxin stimulation model support the difference in gene expression between monosomy and disomy for the X chromosome. Considering the enhanced inflammation in females, cytokine production may be assumed to be higher in females than males. Even if all results are not clear-cut, nonetheless, many studies have reported higher cytokine levels in both male humans and animals than in females. High IL-6 levels in males correlated with poorer prognosis and shorter longevity. A sound understanding of the basic regulatory mechanisms responsible for these gender differences may lead to new therapeutic targets.

## Review

### Definition of inflammation

In the majority of cases, inflammation is a defense mechanism of sudden onset that restores homeostatic balance in the case of infection, tumor, and traumatic or ischemic tissue damages. Vascular and exudative processes are predominant with respect to the clinical signs. They are the expression of a protective tissue response to injuries, which serves to destroy, dilute, or wall off both the injurious agent and the injured tissues.

Chronic inflammation is less frequent and is mainly observed in cases of prolonged and persistent inflammation predominately marked by new connective tissue formation. It tends to persist for several weeks, months, or years, with a vague and indefinite termination. Chronic inflammation occurs when the injuring agent (or products resulting from its presence) persists in the lesional area, and when the host's tissues respond in a manner (or to a degree) that is insufficient to completely overcome the continuing effects of the injuring agent. Histopathologically, chronic inflammation is characterized by infiltrates of lymphocytes, plasma cells, histiocytes, and fibrosis. Local deleterious effects are usually observed in the affected tissues leading to permanent organ damages that compromise the disease's prognosis.

At the clinical level, the intensity of inflammation is measured using inflammatory markers, such as C-reactive protein (CRP), erythrocyte sedimentation rate (ESR), or neutrophil count. Higher marker levels are only an indication of the importance of the inflammatory phenomenon.

### Expression of biological markers underlying inflammatory processes

When analyzing these standard inflammatory biological markers, significant differences were observed not only in adults [1] but also between boys and girls younger than 10 years old. Biological parameters increased at the beginning of the inflammatory process and over time, and CRP, ESR, and neutrophil counts reached or exceeded threshold levels, with values being systematically higher in girls than those in boys. However, at the peak inflammatory response, the values observed were rather similar [2]. Neutrophil counts were reported to be higher in females of all ages in several acute inflammatory conditions, such as post-surgical trauma following gastrectomy [3], exercise-induced muscle injury [4], coronary artery diseases [5], children born to hepatitis C-infected women [6], and even in several basic conditions [7]. Women who subsequently developed cardiovascular events were shown to display higher baseline CRP levels than males of corresponding status [8]. CRP values were also higher in women participating in the Dallas heart study [9]. Females tended to exhibit higher baseline ESR [10], even during childhood [2], which was also observed in pathologic conditions, such as rheumatoid arthritis [11]. The question is, therefore, whether these sex differences in terms of biological markers are associated with clinical differences.

### Sexual dimorphism in respiratory diseases along the life course

During the 1970s, hyaline membrane disease-related mortality was higher in boys than that in girls, with ratios ranging from 1.62 to 1.76 in a published meta-analysis [12]. As regards the meconium aspiration syndrome, male neonates were also more prone to develop the disorder than female neonates [13]. Recent data on 92,332 patients admitted to pediatric hospitals demonstrated a consistent excess of males over females (59%) for almost all disease categories, with 26% of diseases relating to the respiratory system. Boys were also more susceptible than girls to acute viral infections, such as bronchiolitis (61% vs. 53%, *P* = 0.01) [14]. In a survey involving 7,001 tuberculosis patients from Bangladesh, the female/male ratio was found to be 0.33:1 [15]. The tuberculosis case notification rate was reported to be higher for men than that for women worldwide [16]. In 2013, tuberculosis epidemiology is still characterized by significant differences in prevalence rates between men and women worldwide, with cases among men exceeding those found in women with a male/female ratio of 2:1 found in some regions [17]. At any age, boys outnumber girls in acute inflammatory diseases, and their prognosis is worse. Better prognosis in females for acute inflammatory diseases is observed regardless of the age, while worse outcome concerns females of every age with chronic inflammation. Compared to age, the chronicity of the inflammatory process has more adverse effects on girls than on boys by its collateral perverse consequences. When inflammation is not reversible, when the power “ON/OFF” switch of inflammation remains continuously moved to the ON position, the largest inflammatory mobilization in girls is responsible for deleterious effects. The longer of the inflammation of a tissue lasts, the more damage it does. That could explain the higher mortality in girls suffering from cystic fibrosis or chronic obstructive pulmonary disease (COPD) (Table 1).

**Table 1:** Worse prognosis in males in acute diseases and in females during chronic inflammatory diseases

**Disease**	**Number of patients**	**Age of patients**	**Significance**	**References**
Acute diseases (higher in males)				
Hyaline membrane disease	54,064	Premature newborns	Ratio 1.76:1	[12]
Meconium aspiration	21,472	Newborns	*P* < 0.02	[13]
Mortality and morbidity of hospitalized children	Nearly 500,000	Children	*P* < 0.001	[18]
Severe sepsis in children	1,586,253	Children	*P* < 0.001	[19]
Pediatric hospital admissions	92,332	Children	Males 59%	[20]
Acute respiratory failure after bone marrow transplantation	318	Children	*P* < 0.04	[21]
Tuberculosis in Bangladesh	266,189	Adults	Ratio M/F, 1:0.33	[15]
Tuberculosis in India	90,000	Adults	*P* < 0.05	[16]
Community-acquired pneumonia	2-year prospective study	Adults	*P* < 0.0001	[22]
Acute respiratory distress syndrome	333,004	Adults	*P* < 0.01	[23]
Post-injury pneumonia	30,288	More than 65 years	*P* < 0.001	[1]
Hospitalized community-acquired pneumonia	623,718	More than 65 years	*P* < 0.001	[24]
In-hospital community-acquired pneumonia (elderly)	717	More than 65 years	*P* < 0.02	[25]
Australian seniors with chronic respiratory disease	108,312	More than 65 years	*P* < 0.0003	[26]
Mycoplasma respiratory disease (mice)	55	Adult animals	*P* < 0.001	[27]
Chronic respiratory diseases (poorer prognosis in females)				
Chronic inflammatory diseases (children)	149 children	Children	*P* < 0.01	[28]
Asthma in adolescents (sex ratio in severe asthma 5 F/1 M)	2,693	Adolescents	Ratio 1.85:1	[29]
Chronic pulmonary diseases (females smokers)	Meta-analysis (55,709)	Adults	*P* < 0.008	[30]
HIV-related pneumocystis pneumonia	3,070	Adults	*P* < 0.001	[31]
Cystic fibrosis	21,047	1 to 20 years: females 60% more likely to die, outside same risk	*P* < 0.001	[32]
Asthma	3,013	5 to 62 years	*P* < 0.01	[33]
Chronic obstructive pulmonary diseases (COPD) emergencies	794	Adults	*P* < 0.01	[34]
COPD	Clinical commentary	Adults	More females than males	[35]

In a 2-year prospective study covering a well-defined geographic area of the Spanish Mediterranean coast, the incidence rates of community-acquired pneumonia were higher in males (16 vs. 9 cases per 10,000 people per year, *P* < 0.0001), in particular among patients aged >75 years, with 87 cases per 10,000 people found per year in this patient population [22].

In addition, in animals, there was a male/female bias in relation to induced occurrence, as male mice consistently developed a more severe disease in the lung parenchyma infected by *Mycoplasma pulmonis* as compared to females [27].

Male gender was reported to be associated with an increased risk of pneumonia and poorer pulmonary prognosis in various clinical conditions, including post-injury and hospitalized community-acquired pneumonia, acute respiratory distress syndrome, HIV-related *Pneumocystis carinii* pneumonia, current respiratory illness in children, and acute respiratory failure following bone marrow transplantation, along with a higher risk of re-hospitalization [1,21,23-25,31,36]. In many acute respiratory diseases, males appear, as they age, to be more susceptible to complications and exhibit a higher mortality [26]. In 1965, an international literature review by Washburn et al. revealed a significant male preponderance for respiratory diseases, especially during infancy [37]. A genetic hypothesis was proposed, suggesting that a gene locus on the X chromosome in humans was involved in the synthesis of immunoglobulins. The male preponderance and poorer prognosis status were found not only for respiratory diseases but also for most acute inflammatory processes. Several studies from various countries suggested that males, children as well as adults, were more frequently admitted to intensive care units than females and, thus, more likely to require aggressive life support [18,38]. In a recent work on predictors of sequelae and death following bacterial meningitis in childhood, male gender was reported to be a significant prognostic factor, with no satisfactory explanation found [39]. Even in animals, infectious diseases [40] and myocardial infarction [41] were associated with poorer prognosis in males than in females. One possible explanation for this is that inflammatory reactions are likely to be driven by hormonal status. Nonetheless, this prognostic difference was even observed in preterm infants, as documented in several papers [19,42]. Clinical data from prepubertal children suggested that potential gene expression differences depended on sex chromosomes rather than hormonal status. Indeed, in these children, hormonal status was largely immature, and sexual hormones were far less abundant, although some studies have shown that in very young children, the mean estrogen levels—still considerably below adult levels—were eight times higher in girls than those in boys [43]. Therefore, in prepubescent children, hormonal differences may still influence the inflammatory response.

### Higher inflammatory response in girls

Although the inflammatory response was reported to be higher in females than in males with acute or chronic respiratory diseases [30], the consequences of severe inflammation varied, depending on the inflammatory process. Females were shown to display a protective advantage in acute conditions, whereas they were more likely to present deleterious tissue damage in relation to continuous chronic inflammation. Gender differences in terms of clinical outcome indicate that during early childhood, chronic inflammatory events underlying the physiological disease mechanism may be more damaging in females than in males. These observations may explain the results of a large American study involving more than 21,000 patients aged 1–20 years that revealed a gender gap in cystic fibrosis mortality [32], with girls exhibiting a 60% higher mortality risk than boys. This difference could not be accounted for by other risk factors, such as nutritional status, pulmonary function, pathogens in sputum, pancreas insufficiency, or age at diagnosis. In another study relating to asthma [29], severe cases were more frequent in females and more persistent in adulthood, with higher mortality rates after 40 years of age. At any age, severe asthma concerns about 5% of all asthma cases, but females are almost five times more likely than males to belong to this category, even if there is a male preponderance when considering the whole cohort of asthmatics, with bronchial hyperreactivity shown to be more common in girls, and atopy more common in boys [44]. In addition, new data from a North American population study revealed that males were 46% less likely to experience asthma exacerbation than females (odds ratio 0.54, 95% confidence interval 0.31–0.94) [33].

Recently, female gender was associated with poor prognosis of COPD and higher need for post-hospital support [34]. COPD disease expression was found to be different in women and men. In addition to its increased prevalence in females in the case of non-smoker COPD, with patients being more likely to be female, women were more prone to have an airway-predominant (chronic bronchitis) phenotype, and men more likely to develop an emphysema phenotype. This observation was considered to be the consequence of genetic differences in the way males and females manifest damage. Women experienced more severe dyspnea and anxiety, as well as lower disease-related quality of life. Furthermore, when discontinuing treatment with inhaled corticosteroids, women presented a greater likelihood of respiratory deterioration in comparison with men [35]. In a recent paper, we reported that chronic inflammatory diseases in children were more severe in girls than in boys [28]. Our data in the prepubescent children hospitalized for severe exacerbations of chronic asthma supported the hypothesis that inflammation was enhanced in girls, requiring higher corticosteroid doses, longer duration of inhaled therapy, and prolonged hospital stays. In cystic fibrosis as well, inflammation was more marked in females, which was also the case for sickle cell anemia, where we revealed, in line with other authors, that the unexplained poorer prognosis of females was related to a lower forced vital capacity [45].

### Hormonal influences

While estrogens exhibit diverse effects on inflammation and immune responses, the molecular basis of these effects is poorly understood. It is possible that fluctuations in estrogen levels modulate intracellular signaling for immune responses via estrogen receptors. Based on recent a data, 17beta-estradiol was shown to increase, at physiologically high concentrations, the activity of NF-kappa B in human T cells and induced T cell survival, which may possibly account for the observed gender dimorphism [46]. However, there are other studies showing that estrogens can downregulate the NF-kappa B signaling pathway [47]. A further study revealed that female sex hormones (salutary effects) and male sex steroids (suppressing cardiac and immune functions) were likely to influence the outcome of trauma patients, although the exact mechanisms were not clearly understood [48]. There is still an unresolved paradox with respect to estrogens regarding their immunosupportive role in trauma/sepsis and proinflammatory effects in certain chronic autoimmune diseases in humans. The Straub review reinforced the concept that estrogens exhibit anti-inflammatory and proinflammatory effects, although no uniform concept covering all inflammatory conditions could be found because of the highly variable responses of the immune and repair systems [49].

Recent studies focused on the more robust behavioral and somatic responses to stress observed in females, and on the latter's more potent immune and inflammatory reactions [50]. Testosterone has been reported to be inversely associated to the inflammatory process, especially to the levels of markers such as C-reactive protein [51]. Numerous studies evaluated receptors, cytokine production, and clinical outcomes in both animals and humans, revealing that estrogens clearly modulate the immune response. However, these observations were not sufficient to readily explain the universal gender difference observed in acute inflammation, which was found across all age groups, from premature infants to geriatric patients.

### Cytokine expression

In view of the enhanced inflammatory reactions in females, cytokine production may be assumed to be higher in females than in males. Some studies did in fact confirm higher cytokine levels in females, especially in the context of estrogen stimulation [52]. However, other studies reported that IL-6 levels did not change during the menstrual cycle [53]. Although estrogens were shown to be able to slightly modulate cytokine levels, the variations observed were usually not significant or only approached significance when considering stimulations at various periods of the menstrual cycle. In most cases, however, and all along the life course, cytokine concentrations in response to inflammatory stimulation were shown to be higher in males than in females. Estrogen may exhibit pro- and anti-inflammatory properties depending on the context and the involved tissue. Several studies have reported a significantly higher cytokine production in males [54,55], using *ex vivo* models in humans [56-59] and animals [60-62]. On admission to the emergency department, biomarker patterns in men were characterized by higher levels of tumor necrosis factor, interleukin (IL)-6, and IL-10, which were associated with poorer 1-year survival rates, as reported by the *Genetic and Inflammatory Markers of Sepsis Investigators Group*[54]. In a wild-type mice model using intraperitoneally injected endotoxin, males displayed stronger responses than females with respect to myeloid differentiation, myeloid cell release from bone marrow into peripheral blood, and migration into the inflammatory peritoneal cavity, demonstrating that sex differences in inflammatory responses were also observed in animals [55] (Table 2).

**Table 2:** Biological and clinical features associated to sexual dimorphism in inflammation

	**Inflammatory mediators**	**Female**	**Male**
Biological markers	CRP	⬆	⬇
	ESR	⬆	⬇
	IL-8	⬆	⬇
	IL-1β, IL-6, TNF-α	⬇	⬆
	CD99	⬇	⬆
Hematological markers	Neutrophil count	⬆	⬇
	Monocyte count	⬇	⬆
Clinical features	Outcome in acute inflammatory processes (septic shock, burns, trauma)	Better prognosis	Worse prognosis
	Outcome in chronic inflammatory processes (severe asthma, cystic fibrosis, BPCO)	Worse prognosis	Better prognosis

The table summarizes the main trends observe in the literature. Up arrow denotes increase; down arrow, decrease.

In our first *in vitro* study, we investigated the influence of sex on the production of cytokines involved in inflammation by studying healthy prepubescent males and females, as well as Turner syndrome patients with an XO genotype (X monosomy) [63]. In this model, despite all Turner syndrome patients being female, there was a male pattern of reactivity, with cytokine production response to endotoxin and pokeweed mitogen (PWM) stimulation of whole blood cells being higher in males than that in females, suggesting a genetic-chromosomal influence. Thus, these findings contrast the numerous clinical observations revealing *in vivo* increased inflammatory marker production in females. However, in our stimulation model, sex differences in cytokine production depended on the type and intensity of the stimulus (0.2 or 1 ng/ml of endotoxin or PWM), and varied according to the cytokine (IL-1, IL-6, TNF-α, or PGE-2). We observed that, in both males and females, TNFα was highly correlated to IL-6, provided that low doses of lipopolysaccharide or PWM that induced responses in approximately the same relative range were used. A strong correlation between IL-6 and IL-1 was found, but only in the male group. Given the potential anti-inflammatory effect of IL-6 [64], these relationships may reflect a regulation of both cytokine levels by concomitant IL-6 production. Furthermore, males displayed a significantly lower variation coefficient (slope) than females, suggesting that the influence of IL-6 in reducing IL-1 or TNFα production was more pronounced in males. Caution must be taken, however, as the interindividual variations observed in this study were accounted for by a potential intraindividual modulation inherent to this model, which has not been confirmed to date. Gregory et al. investigated sex differences in cell-mediated immunity in both normal and pathological conditions, following dorsal scald or sham injury in mice [65]. The authors observed differences in both delayed-type hypersensitivity and splenocyte proliferative responses between males and females. These responses were significantly suppressed, with a kinetic profile depending on sex. There was a high expression of IL-6 at the end of the suppression, while anti-IL-6 antibodies were shown to restore splenocyte proliferation, yet only partially in males but completely in females. This data demonstrates that cell-mediated immunity varies according to sex and that the response to the same inflammatory stimulation differs in males and females, and this is not only in terms of kinetics (quantitatively and qualitatively) but also due to the complex relationships between inflammatory mediators.

In the lungs, complex cytokine networks have been observed, whereby inflammatory cells were shown to regulate fibroblast function, which in turn regulates inflammatory cell function. The effect of an individual cytokine varies according to the activation status of the target cell, presence of other cytokines in the local microenvironment, and ability of the target cell to produce bioactive autacoids, such as prostaglandins [66]. Another study revealed that the inflammatory response, especially to endotoxins, might be predicted by several parameters, such as the promoter polymorphism of the IL-6 gene, sex with males displaying enhanced IL-6 levels as compared to females (*P* < 0.015), density of the endotoxin receptor CD14, and finally, the concentration of other cytokines. In addition, the IL-6 response correlated with the concentrations of the more proximal cytokines, namely tumor necrosis factor-α and interleukin-1β [67].

While age may influence the production of cytokines, sex differences remain apparent [68]. Considering the poorer prognosis of males and their higher IL-6 blood levels, Bonafè et al. investigated 700 subjects aged 60–110 years, including 323 centenarians [69]. The authors showed that the IL-6 promoter genetic variability at the −174 C/G locus and its effect on IL-6 serum levels clearly influenced longevity in homozygous men. The proportion of subjects homozygous for the G allele at the −174 locus decreased in centenarian males, but not in centenarian females. Higher levels of inflammatory markers were predictive of reduced survival time and shorter lifespan among men, whereas only IL-6 at high levels was associated with longevity in post-menopausal women who did not take estrogens [70].

### X-linked genes involved in inflammation

Several proteins involved in immunity are encoded on the X chromosome and especially protein members of the toll-like receptor signaling pathway, glucose metabolic enzymes, apoptotic cascade enzymes, or proteins with impact on the hormonal balance. Females carry two X chromosomes, one of which is randomly inactivated during early embryogenesis in order to maintain the dosage balance of proteins for both sexes [71-73]. Females are, thus, a mosaic of cells with genes from either the paternal or maternal X chromosome. Therefore, polymorphism of X-linked genes would result in the presence of two cell populations with distinct regulatory and functional arsenals, providing females with greater diversity to fight against infectious challenges, in comparison with the uniform cell populations in hemizygous males [74-76]. Moreover, up to 15% of the X-linked genes escape inactivation to some extent, thereby increasing the X-linked cellular protein content in females compared with that in males [77,78]. Some of the main protein members of the toll-like receptor (TLR) and nuclear factor κB (NF-κB) signaling pathway are linked to the X chromosome: interleukin 1 receptor Y-associated kinase 1, NF-κB essential modulator, and Bruton's tyrosine kinase.

Chandra et al. used an animal model to investigate whether female X-chromosome mosaicism for inflammatory gene expression may contribute to the gender dimorphic response during the host response. Using splenic T-cell depletion and post-endotoxin IL-10 responses, the authors showed that the inflammatory response in mosaic animals did not simply display an average of the deficient and wild-type responses, but that the mosaic subjects exhibited a unique characteristic response [79]. In another study, the authors showed that cellular mosaicism for the X-linked gp91phox (NOX2) deficiency, the main subunit of the superoxide anion-generating NADPH oxidase complex, reduced sepsis-induced mortality in deficient mice, as well as in mosaic animals carrying both deficient and WT phagocytes. In mosaic animals, the deficient neutrophils displayed increased organ recruitment and CD11b membrane expression compared with WT neutrophils [80].

In our first *in vitro* study, the similarities between the male subjects and Turner syndrome patients in terms of the cytokine production pattern support a difference in gene expression between monosomy and disomy for the X chromosome [63]. More recently, we sought to evaluate the potential role of the X mosaicism on the expression of the TLR signaling pathway by measuring the production of inflammatory cytokines through the NF-κB pathway in healthy adults using whole blood stimulated by a gram-negative bacillus endotoxin [81]. In this study, we also observed a higher production of tumor necrosis factor (TNF)-α, with a trend towards a higher IL-6 production, in men. TNF-α production significantly correlated with monocyte counts, with men exhibiting a higher monocyte count than women. One of our hypotheses is that, in males, an early release of high TNF-α and IL-6 levels by monocytes may produce hyperinflammation, thereby contributing to the higher mortality in septic shock observed in males versus females.

In autoimmune diseases [82], immune response genes were shown to be significantly over-represented among genes upregulated in women and among the immune response genes, with the inflammatory/cytotoxic effector genes interferon-γ, lymphotoxin β, granzyme A, interleukin-12 receptor β 2, and granulysin being among those overexpressed to the greatest extent. In contrast, interleukin-17A was the only effector gene found to be more strongly expressed in men.

Another particularity of the X chromosome, which may be involved in the immune sex dimorphism, is the spreading of the inactivation signal along the pseudoautosomal (PAR1) region of the X chromosome. The accidental methylation of this region may result in partial silencing of genes lying close to the boundary between the PAR1 region and X-linked material. In our study, the percentage of the monocytes expressing CD99 was higher in men than that in women, thus confirming the higher CD99 expression reported in males using reverse transcription polymerase chain reaction [71]. CD99 is known to play a role in leukocyte diapedesis, and its gene lies closest to the boundary between the PAR1 and X-linked regions. Consequently, a difference in the percentage of monocytes expressing CD99 could affect monocyte recruitment to the inflamed tissues. Increased CD99 expression due to an X;Y translocation was linked to the early development of a chronic inflammatory disease known to be associated with an imbalanced sex ratio [83]. Some genes implicated in the immune response and located on the X chromosome, such as X-linked genes encoding the proteins of the TLR signaling pathway or genes of the pseudoautosomal PAR1 region such as the CD99 gene, could be responsible for some of the sex-specific responses to inflammation. Therefore, as mosaicism for X-linked polymorphisms may be of clinical relevance, this should be considered in genetic association or sex-related clinical studies.

## Conclusions

The investigation of the inherent biological advantages of healthy normal females may lead to new therapeutic strategies aimed to improve anti-infectious responses in males. Anti-inflammatory drugs exert differing effects on both animals and humans depending on gender [84-86]. Sex may influence the potency of test compounds, such as steroids, or the doses to be used; sex may also determine the phenotype answer to specific drugs. In a 2003 study involving 60,694 Californian asthmatic subjects, females aged 14 to 22 years were found to be more likely outpatients or visit emergency departments and use more oral corticosteroids compared to males [87]. Although females with chronic inflammation used more corticosteroids than males, their prognosis was poorer. Even in animals, chronic inflammation was more significant in females [88]. Accordingly, a better understanding of the inflammatory mechanisms involved in chronic inflammatory processes may be instrumental in decreasing deleterious tissue damage and, thus, be of great benefit for women.

## Competing interests

The authors declare that they have no competing interests.

## Authors' contributions

GJC and JD drafted the manuscript. GJC, FC, JD, and NL participated in the design of the review. GC and JD contributed to the conception of the review. All authors read and approved the final manuscript.

## Authors' information

GJC is a professor of Pediatrics at the Department of Pulmonology and Cystic Fibrosis, Université Libre de Bruxelles (ULB) and the chief medical officer of the University Children Hospital Queen Fabiola, Brussels; FC is the director of the Laboratory of Immunology at the Brugmann University Hospital, Brussels. JD is a professor of Immunology, Laboratory of Pediatrics (ULB). NL is a pediatrician and a doctoral degree student (ULB).
